# Neurological manifestations of post-COVID-19 syndrome S1-guideline of the German society of neurology

**DOI:** 10.1186/s42466-022-00191-y

**Published:** 2022-07-18

**Authors:** Christiana Franke, Peter Berlit, Harald Prüss

**Affiliations:** 1 Department of Neurology and Experimental Neurology, Charité Universitätsmedizin Berlin, Hindenburgdamm 30, 12200 Berlin, Germany; 2 Secretary General of the German Society of Neurology, Berlin, Germany; 3 German Center for Neurodegenerative Disorders (DZNE), Berlin, Berlin, Germany

**Keywords:** Long-COVID, Post-COVID-19 syndrome, Cognitive deficits, Headache, Myalgia, Neuropathy, Vertigo, Hyposmia, Hypogeusia

## Abstract

Infection with the severe acute respiratory syndrome coronavirus 2 (SARS-CoV-2) leads to COVID-19 (COrona VIrus Disease-2019). SARS-CoV-2 acute infection may be associated with an increased incidence of neurological manifestations such as encephalopathy and encephalomyelitis, ischemic stroke and intracerebral hemorrhage, anosmia and neuromuscular diseases. Neurological manifestations are commonly reported during the post-acute phase and are also present in Long-COVID (LCS) and post-COVID-19 syndrome (PCS). In October 2020, the German Society of Neurology (DGN, Deutsche Gesellschaft für Neurologie) published the first guideline on the neurological manifestations of COVID-19. In December 2021 this S1 guideline was revised and guidance for the care of patients with post-COVID-19 syndrome regarding neurological manifestations was added. This is an abbreviated version of the post-COVID-19 syndrome chapter of the guideline issued by the German Neurological society and published in the Guideline repository of the AWMF (Working Group of Scientific Medical Societies; Arbeitsgemeinschaft wissenschaftlicher Medizinischer Fachgesellschaften).

## Introduction

The global confirmed case count of coronavirus disease 2019 (COVID-19) reached 500 million as of April 2022 (https://coronavirus.jhu.edu/map.html). In Germany 23 million patients are officially reported as affected by COVID-19. The actual case positive rate is estimated to be much higher with various models predicting the actual number to be much higher than the number of confirmed cases [15, 36]. Approximately 10–40% of COVID-19 patients with mild acute infection report of residual or new symptoms as sequelae [29], and up to 80% of patients hospitalized for COVID-19. The global prevalence of post COVID-19 symptoms in a recent meta-analysis was 43%, with 54% after hospitalization and 34% in outpatients [11].

The clinical presentation includes a wide range of unspecific symptoms affecting all organ systems [24] and predominantly occurring in female patients [6, 10, 28].

The frequently missing control group in the data published to date carries the risk of overestimating the incidence of LCS. A French cross-sectional study showed that people who have not been infected with SARS-CoV-2 report residual symptoms with equal frequencies compared to those with LCS after confirmed infection [25]. The disease surveillance by the British "Office for National Statistics" shows that the broad spectrum of symptoms described for PCS also occur to a relevant extent in control subjects who did not have COVID-19 (https://www.ons.gov.uk/peoplepopulationandcommunity/healthandsocialcare/conditionsanddiseases/articles/technicalarticleupdatedestimatesoftheprevalenceofpostacutesymptomsamongpeoplewithcoronaviruscovid19intheuk/26april2020to1august2021). 5.0% of the patients reported having at least 1 of 12 defined symptoms 12–16 weeks after the SARS-CoV-2 infection, compared to 3.4% in the control group. In children up to the age of 11, the symptoms occurred even more frequently in the non-infected control group (4.1% versus 3.2%). When patients requiring hospitalization because of SARS-CoV-2 infection are compared after 6 months with matched control patients hospitalized for non-COVID-19 illness neuropsychiatric outcomes were not different with the exception of anosmia, which was 4 times more frequent after COVID-19 [26].

Diagnostic work up and treatment of affected patients should be performed in an interdisciplinary setting consisting of cardiologists, pneumologists, psychiatrists/psychosomatic doctors and neurologists.

### Definition

Clinical symptoms occurring during or residually after COVID-19 are defined in relation to the time interval following the acute infection. The post-acute phase includes the period up to 4 weeks after the acute infection. LCS includes clinical symptoms more than 4 weeks after COVID-19 (https://www.nice.org.uk/guidance/ng188, https://www.who.int/publications/i/item/WHO-2019-nCoV-Post_COVID19_condition-Clinical_case_definition). PCS is present when clinical symptoms occur during or after an illness compatible with COVID-19, persisting for at least 2 months, fluctuating in occurrence and cannot be explained by any other diagnosis. The acute infection dates back at least 12 weeks. Affected are patients who have gone through COVID-19 with a mild to moderate course and remained in quarantine at home, as well as patients who had to be admitted to hospital or were treated on intensive care unit (ICU) [2, 30].

### Neurological manifestations

The most common neurological complaints are fatigue, concentration and memory disorders, headache, vertigo, myalgia and neuropathy, as well as persistent smell and taste disturbances [8, 17, 35]. Autonomic dysregulations have also been described [23]. The complaints can differ in intensity, fluctuate strongly and may interact with other stress factors. An improvement of residual symptoms occurs in a large number of patients without specific treatment within the first 12 weeks after the acute infection.

Additionally, neurological diseases are reported as sequelae after COVID-19. The risk of (cerebro-)vascular disease, including stroke and transient ischemia, is reported to be approximately 50% higher within the first year after the acute infection with SARS-CoV-2 [37]. Epileptic seizures, myelitis, but also peripheral neurological diseases such as Guillain-Barré syndrome (GBS), cranial nerve deficits, myositis and plexopathies have been described [3, 7, 9, 38]. Also, rare cases of autoimmune encephalomyelitis were reported 3 months after COVID-19 [22].

## Post-COVID-19 associated symptoms, diagnostics, and therapy in detail

### Cognitive disorders and fatigue

Cognitive deficits found both in the subacute stage and in the further course after COVID-19 comprise executive functioning, processing speed, category fluency, memory encoding and recall. This applies to patients with both initially mild and severe COVID-19 courses [1, 4, 27]. Cognitive deficits are also reported to coexist with fatigue [34], which commonly leads to severely limiting, disproportionate, subjective exhaustion on a somatic, cognitive and/or psychological level. Postexertional malaise and exertion intolerance, not sufficiently modulated through sleep or recovery are key features of fatigue.

If self-reported cognitive deficits occur, a cognitive screening assessment e.g. the Montreal Cognitive Assessment (MoCA) should be performed. If pathological results are detected and the symptoms are persistent for several months with restrictions regarding the activities of daily life (ADLs), further examination is indicated. Diagnostic includes the examination of serum and cerebrospinal fluid (CSF) including CNS autoantibodies against intracellular and surface antigens and neurodegenerative markers, cerebral imaging, and detailed neuropsychological assessment. A significant association between neurocognitive symptoms and anti-nuclear antibodies (ANA) may indicate autoimmunity as an etiological cofactor in post-COVID-19 syndrome [33]. Antineural autoantibodies in CSF are associated with pathological performance in cognitive screening assessment (Franke et.al. manuscript currently under revision). Mannose-binding lectin and increased levels of interleukin 8 have been described as possible biomarkers but are not yet used routinely [21].

Self-report questionnaires such as the Fatigue Scale (FS), the Fatigue Severity Scale (FSS) or the Fatigue Assessment Scale (FAS) should be used to assess the symptoms and severity of fatigue.

To date there are no established and effective medical treatment options for post-viral fatigue and cognitive impairment, as well as related conditions such as myalgic encephalomyelitis/Chronic Fatigue Syndrome (ME/CFS). If there are indications of autoimmunity, therapeutic approaches including corticosteroids, intravenous immunoglobulins or therapeutic apheresis can be administered depending on risk and expected benefit.

A causal therapy for fatigue is unknown. Non-drug therapeutic approaches such as relaxation techniques, moderate physical activity, acquisition of adequate coping behavior strategies in addition to psychotherapeutic or psychopharmacological treatment are recommended.

### Headache

A meta-analysis of cohort studies indicates that headache persists in 44% after COVID-19 illness. If headache is already reported during the acute infection, there is an increased prevalence of persistent headache in the context of post-COVID-19 [14].

Self-report instruments (e.g. Brief Pain Inventory) should be used to assess symptoms including the severity of chronic pain. Depending on the clinical presentation and examination, an extended laboratory examination can be carried out to rule out other (e.g. rheumatological) causes. MR imaging should be performed in individual cases to exclude structural causes.

To date headache related to COVID-19 is treated in analogy to chronic tension headache. Prophylactic treatment options including amitriptyline are recommended. Therapeutic approaches follow the existing guidelines of the German Society of Neurology (DGN) (https://dgn.org/leitlinien/ll-56-ll-therapie-des-episodischen-und-chronischen-kopfschmerzes-vom-spannungstyp/).

### Hyposmia/anosmia and hypgeusia/ageusia

A reduction or loss of smell and taste is frequently reported in the aftermath of COVID-19 and may last longer than 6 months after the acute infection [2].

Hyposmia/hypgeusia or anosmia/ageusia should be objectified e.g. using the SS-16 item sniffin sticks test and taste test. Neurological and/or ENT presentation should include a thorough case history excluding competing or alternative causes. Further laboratory diagnostic and endoscopy may be considered. In addition, atrophy of the olfactory bulb has been reported [20]

Constant and structured olfactory training is recommended [12]. The aim is to stimulate the regeneration of olfactory receptor neurons of the olfactory mucosa. The odor of rose, lemon, eucalyptus and cloves are commonly used (Hüttenbrink KB, et al. Riech- und Schmeckstörungen. S2k-Leitlinie der DGHNOKHC. Stand: 31.10.2016. AWMF online). The application of intranasal corticosteroids has been reported in case reports but remains controversial [18].

### Myalgia, muscle weakness and neuropathy

Muscle pain, particularly of the proximal muscles, and muscle weakness are commonly reported and may persist for up to 6 months after the acute infection [17, 30].

Subsequent to case history and neurological examination, a laboratory investigation of blood including sedimentation rate, myoglobin, creatine kinase and myositis antibodies if clinically suspected, as well as examination of CSF are recommended. An electrophysiological examination is indicated. These recommendations are in line with existing guidelines regarding muscular and neuromuscular disorder (https://dgn.org/leitlinien/ll-030-067-diagnostik-bei-polyneuropathien-2019/, https://dgn.org/leitlinien/ll-030-051-diagnostik-und-differenzialdiagnose-bei-myalgien-2020/, https://dgn.org/leitlinien/ll-030-115-diagnostik-von-myopathien-2021).

Therapeutic procedures depend on diagnostic findings and are applied according to existing guidelines (https://dgn.org/leitlinien/ll-69-ll-myositissyndrome). If laboratory results obtain unremarkable, symptomatic treatment can be tried, e.g. administering gabapentine or pregabaline. Physiotherapy and moderate exercise should be implemented.

### Pathophysiology of LCS and PCS

Pathophysiological mechanisms in PCS and LCS are scarcely understood. Different explanatory pathways are currently discussed e.g. neurotransmitter-mediated changes, an endothelial-microcirculatory dysregulation, persisting (non-specific) post-infectious inflammation and (virus-triggered) immune-mediated mechanisms including humoral and cell-mediated autoimmunity [31]. In most published studies, SARS-CoV-2 RNA was neither detected in CSF, nor intrathecally produced SARS-CoV-2 directed IgG antibodies can be held responsible as the cause of PCS [32]. Neurofilament, a neuronal degeneration marker is interestingly often increased in patients with neurological manifestation during the acute infection, however not in patients with PCS [19]. 18FDG-PET ([18F]-fluorodeoxyglucose positron emission tomography) conducted in LCS patients with cognitive deficit (less than 26/30 points on the MoCA test; Montreal Cognitive Assessment) showed in 10/15 patients a hypometabolism in frontoparietal brain regions [16]. A follow-up [5] of eight patients six months after the acute infection showed an improvement in symptoms and considerable normalization of brain metabolism in PET.

Longitudinal MRI studies have demonstrated changes in the structure of the prefrontal cortex and parahippocampal regions [13].

Already the SARS-1 pandemic has proven that individual patients may retain long-lasting clinical complaints, especially pain, fatigue, depression, and sleep disorders. The lack of disease-specific biomarkers impedes etiological assignment as well as the exclusion of other (premorbid) diseases.

## Conclusion

Neurological manifestations are common in patients with LCS and PCS; commonly reported are cognitive deficits, fatigue, headache, myalgia and neuropathy. A comprehensive—ideally interdisciplinary—diagnostic assessment should be initiated in patients who complain about residual or new symptoms more than 3 months after the acute infection. If immune involvement is detected, immunomodulatory therapy may be considered as an individual treatment attempt. A causal therapy does not yet exist; the symptomatic therapy is carried out according to the guidelines of the DGN. Structured neurorehabilitation programs are urgently needed. In parallel, patients should be offered concomitant psychosomatic co-treatment. Avoiding SARS-CoV-2 infection is the key most important preventive factor, and therefore vaccination against SARS-CoV-2 with one of the available vaccines is highly recommended. An interdisciplinary treatment including internal, psychosomatic, and psychiatric expertise is crucial in diagnostic and treatment of PCS patients.

## Data Availability

Not applicable.
